# A143 SAFETY OF BIOLOGICAL THERAPIES IN ELDERLY IBD: A SYSTEMATIC REVIEW

**DOI:** 10.1093/jcag/gwab049.142

**Published:** 2022-02-21

**Authors:** P Golovics, G Drügg Hahn, P Wetwittayakhlang, G Wild, W Afif, A Bitton, T Bessissow, P L Lakatos

**Affiliations:** 1 Division of Gastroenterology, McGill University Health Centre, Montreal, QC, Canada; 2 Gastroenterology, McGill University Health Centre, Montreal, QC, Canada; 3 IBD Centre, McGill University Health Center, Montreal, QC, Canada

## Abstract

**Background:**

There was a significant progress in the medical therapy of inflammatory bowel disease(IBD) with the advent of biological compounds, yet patients may experience adverse events(AE): infusion reactions, serious infections and malignancies, understudied in vulnerable patient populations (e.g. elderly).

**Aims:**

Our aim was to perform a systematic review to assess the safety of the biologic therapies in the elderly IBD population.

**Methods:**

Medline databases and conferences proceedings were searched between January 1, 2010, and June 1, 2021. Two reviewers independently evaluated the collected studies based on inclusion and exclusion criteria. Search was focused on IBD/CD/UC, any biological therapy, and adverse events in the elderly. The methodological quality of the included studies was assessed using the Newcastle– Ottawa Scale (NOS). This study was conducted in accordance with the Preferred Reporting Items for Systematic Reviews and Meta-Analyses guidelines (PRISMA).

**Results:**

Our search identified 2885 articles and 12 congress abstracts trough the data base search, finally 14 peer reviewed papers and 3 abstracts met the inclusion criteria. The majority of the studies were retrospective, merging IBD patients, with an age limit of 60 or 65 years for elderly, from Europe or North America. The gender ratio was equal except in the USA veteran database. The identified studies collected safety data on anti-TNF therapy, vedolizumab and ustekinumab. We selected studies with at least 1 year follow-up period. Ranges of AE rates(infliximab/adalimumab 6–39/100patient-years (PY), vedolizumab 6–26/100 PY and ustekinumab 6–18.2/100 PY), infection rates (anti TNF 2.5–31/100PY, vedolizumab 2.6–77/100 PY and ustekinumab 5.2–35.7/100 PY) or infusion/injection reactions (anti TNF 0–14/100PY, vedolizumab 0.9–5/100 PY and ustekinumab 0–2.6/100 PY), were not different among the biological medication.

**Conclusions:**

We report for the first time the comparative safety of biological therapies in elderly IBD patients. Ranges of adverse events or infections were wide but not different among the medications. Current data are insufficient to suggest prioritizing among biologicals in the elderly based on the safety, larger studies in elderly IBD patients are warranted.

**Funding Agencies:**

None

